# *In Vitro* Testing of Ibrutinib-Loaded Electrospun Nanofibers for Potential Use as a Transdermal Patch Material

**DOI:** 10.1021/acsomega.5c12170

**Published:** 2026-02-20

**Authors:** Hilal Fil, Ozan Yesiltepe, Simge Er Zeybekler, Ozge Kozgus, Sevinc Kurbanoglu, Emin Ilker Medine, Dilek Odaci

**Affiliations:** † Department of Biochemistry, Faculty of Science, 37509 Ege University, Bornova, Izmir 35100, Turkiye; ‡ Department of Nuclear Applications, Institute of Nuclear Sciences, Ege University, Izmir 35100, Turkiye; § Ankara University, Faculty of Pharmacy, Department of Analytical Chemistry, Ankara 06560, Turkiye

## Abstract

Breast cancer is a malignancy that originates in the epithelial cells of breast tissue, presenting a considerable challenge to the physical and mental health of women worldwide. This disease remains a prominent concern in public health and medical research, necessitating ongoing attention for effective management and treatment. In this study, a novel drug delivery material was developed by using ibrutinib (IBR)-loaded electrospun polycaprolactone/poly-l-arginine (PCL-P­(Arg)) nanofibers (ENs) for breast cancer treatment. The system aims to improve site-specific delivery near breast tissue through localized and controlled drug release, offering potential advantages over conventional administration routes. The fabricated PCL-P­(Arg)/IBR ENs were characterized using Scanning Electron Microscopy–Energy Dispersive X-ray Spectroscopy (SEM-EDS), Fourier Transform Infrared Spectroscopy (FTIR), Brunauer–Emmett–Teller (BET) analysis, a swelling test, and the water vapor transmission rate (WVTR) measurements. Drug release profiles were evaluated under *in vitro* conditions at pH 5.5 (the endosomal pH of cancer cells and skin pH range of 4–5.5) and pH 7.4 (the physiological pH). The results showed that adding P­(Arg) significantly increased the ENs’ hydrophilicity, porosity, and swelling capacity. Sustained IBR release was observed at both pH values. The Korsmeyer–Peppas model best explained the release kinetics and demonstrated Fickian and anomalous transport mechanisms. *In vitro* cytotoxicity studies using the MCF-7 breast cancer cell line revealed that PCL-P­(Arg)/IBR ENs significantly reduced cell viability compared to the control group (IBR-unloaded ENs). These findings suggest that PCL-P­(Arg)/IBR ENs provide controlled release, favorable physicochemical properties, and targeted cytotoxicity, representing a promising patch for the treatment of breast cancer.

## Graphical abstract

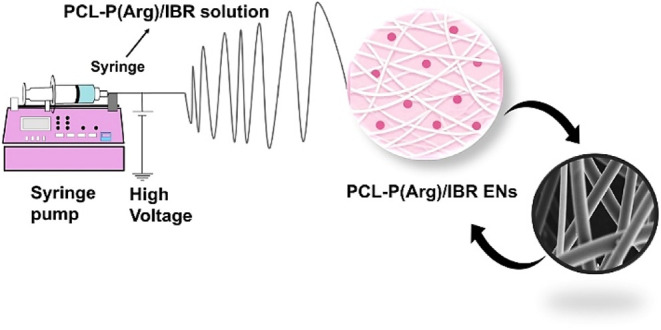

## 1 Introduction

Breast cancer is a malignant condition that develops in the epithelial cells of breast tissue and continues to pose a serious challenge to women’s physical and psychological well-being worldwide. It can also be life-threatening if not diagnosed and managed in a timely manner. The main therapeutic approaches currently employed include surgical interventions (such as lumpectomy and mastectomy), radiation therapy, chemotherapy, and hormonal treatments. However, these conventional clinical procedures are often invasive, exhibit limited specificity, and are commonly associated with substantial adverse effects. Projections estimate that by the year 2050, the number of newly diagnosed breast cancer cases among women will reach around 3.2 million annually. This alarming prediction emphasizes the widespread prevalence of breast cancer, its profound global impact, and the critical necessity for more effective preventive and therapeutic interventions.^1^ The need for effective and site-specific drug carriers is increasing day by day. For this purpose, many nanoscale carrier systems based on nanomedicine applications are available in the literature. Among them, nanofibers are interesting nanomaterials.

Electrospun nanofibers (ENs), produced from polymer solutions via the electrospinning technique in a strong electric field, are gaining recognition as effective transdermal drug-delivery vehicles due to their enhanced characteristics, including structural similarity with the extracellular matrix (ECM), high adsorption capacity, porous structure, ease of drug encapsulation, successful delivery of drugs to tumor sites, decreased burst release of drugs, and biocompatibility.^2–5^ In addition to these advantages, ENs exhibit remarkable qualities that enhance wound healing, facilitating cell growth, proliferation, and adhesion.^6,7^ Furthermore, their outstanding permeability and absorption capabilities enable them to take in exudate from the wound, creating a moist healing environment.^8^ As a result, ENs play a crucial role in enhancing drug delivery and promoting the intricate process of wound healing.

Polycaprolactone (PCL) ENs exhibit remarkable potential to meet the desired mechanical properties while maintaining favorable biocompatibility and biodegradability.^9,10^ However, the inherent hydrophobicity of PCL surfaces poses significant challenges, as it can reduce bioactivity and potentially lead to cytotoxic effects.^11^ To tackle these concerns, a hydrophilic polycationic poly-l-arginine (P­(Arg)), enriched with functional groups, can be used as a promising enhancer for drug delivery.^12,13^ P­(Arg) can interact effectively with the negatively charged skin barrier, facilitating enhanced drug permeation.^13,14^ This approach offers a compelling pathway to overcome the limitations of PCL and improve therapeutic outcomes.

Ibrutinib (IBR) is an approved targeted therapy that selectively attacks cancer cells while sparing normal cells, which helps to minimize severe side effects. It functions as a new irreversible inhibitor of Bruton’s tyrosine kinase (BTK), making it a promising option for chemotherapy.^15^ As a targeted agent, IBR functions as a novel irreversible inhibitor of BTK, which suppresses matrix metalloproteinase-9 (MMP-9), an essential protein linked to tumor progression and metastasis, particularly in breast cancer. Thus, IBR-loaded nanomaterials may provide an effective treatment option for breast cancer.^16^

In this study, a drug delivery system was developed using functional PCL-P­(Arg) ENs created through electrospinning to load IBR. The PCL-P­(Arg)/IBR ENs were designed to improve site-specific delivery to breast tissue by enabling localized and controlled IBR release. P­(Arg) was incorporated into the PCL EN matrix primarily to enhance the hydrophilicity and water uptake capacity of the system, thereby facilitating a more controlled and sustained release of IBR. The developed drug delivery system based on PCL-P­(Arg) ENs was characterized using scanning electron microscopy–energy dispersive X-ray spectroscopy (SEM-EDS), attenuated total reflection–Fourier transform infrared spectroscopy (ATR-FTIR), Brunauer–Emmett–Teller (BET), swelling tests, and the water vapor transmission rate (WVTR). IBR’s release behavior and kinetics were examined under the *in vitro* conditions at pH 5.5 (the endosomal pH of cancer cells and skin pH range of 4–5.5) and pH 7.4 (the physiological pH).^17,18^ In addition, the effects of the developed PCL-P­(Arg)/IBR ENs drug delivery system on MCF-7 cells (breast cancer cell line) were evaluated through *in vitro* cytotoxicity studies.

## 2 Experimental Section

### 2.1 Materials

Ibrutinib (IBR) was kindly supplied by TOBIO NOVELFARMA. Polycaprolactone (PCL; average Mn 80 000), poly-l-arginine [P­(Arg), MW 15 000–70 000], and 3-(4,5-dimethylthiazol-2-yl)-2,5-diphenyltetrazolium bromide (MTT) used for cell viability/cytotoxicity testing were purchased from Sigma-Aldrich (St. Louis, USA). Acetone (AC, 58.08 g/mol, 99.8%) was purchased from Merck (Darmstadt, Germany). Formic acid (FA; MW: 46.03 g/mol, 98–100%) was purchased from ISOLAB (Wertheim, Germany). Eagle’s minimum essential medium (EMEM) cell culture media and fetal bovine serum (FBS), l-glutamine, and penicillin/streptomycin supplements were purchased from Gibco. All of the reagents were of analytical grade and used without further purification.

### 2.2 Apparatus

ENs were produced by using a NanoWeb Electrospun 103 (MaviTech, Turkiye), with a New Era pump system (USA) functioning as the syringe pump. The contact angles of the generated PCL-P­(Arg) ENs were measured using an Attension Theta goniometer (USA). SEM-EDS (Thermo Scientific Apreo S, USA) was employed to assess the morphological characteristics and atomic percentage of the PCL-P­(Arg) ENs. ATR-FTIR (PerkinElmer Spectrum Two, for a range of 4000 to 400 cm^–1^, USA) analysis was carried out to examine the functional groups of PCL-P­(Arg) ENs. The BET specific surface area, total pore volumes, and average pore diameter of the PCL ENs and PCL-P­(Arg)/IBR ENs were determined by using a Micromeritics ASAP 2020 analyzer (USA). UV–vis spectrophotometric measurements were carried out by using a Thermo Scientific Multiskan GO (USA) microplate reader.

### 2.3 Fabrication of ENs

PCL-P­(Arg) ENs were fabricated by preparing a polymer solution containing 12.5% (w/v) PCL and 20 μg/mL P­(Arg) in an FA:AC solvent mixture (3:7, v:v), which was stirred until a homogeneous solution was obtained.^19^ PCL/IBR ENs were prepared by dissolving 12.5% (w/v) PCL and 2 mg/mL IBR in the same FA:AC solvent system under identical conditions. For IBR-loaded PCL-P­(Arg) ENs, the polymer solution consisted of 12.5% (w/v) PCL, 20 μg/mL P­(Arg), and 2 mg/mL IBR in FA:AC (3:7, v:v). All polymer solutions were prepared in equal volumes and electrospun under optimized conditions using an applied voltage of 8.0 kV, a tip-to-collector distance of 10 cm, a flow rate of 1.0 mL/h, a temperature of 23 °C, and a relative humidity of 57–65%. The resulting nanofibers were collected on a 256 cm^2^ collector and cut into 2 × 2 cm^2^ pieces for subsequent drug release and *in vitro* cell viability studies.

### 2.4 Swelling Test

The swelling ratios of the PCL/IBR ENs and PCL-P­(Arg)/IBR ENs were assessed by immersing them in 50 mM PBS solutions at pH 5.5, maintained at 37 °C in an incubator. The nanofibers were cut into 2 × 2 cm pieces, and their initial weights were recorded using a precision balance. Final measurements were taken at 0.5, 1, 2, 4, 6, 8, 10, 12, 24, 48, 54, and 72 h. Before the swollen samples were measured, any excess surface water was gently blotted away with filter paper. The swelling ratios were then calculated using the following eq 1:

1 $$\mathrm{Swelling Ratios}=\frac{W_{f}-W_{i}}{W_{i}}$$

where *W*_i_ is the initial weight of the fiber, and *W*_f_ is the swollen weight.^20^

### 2.5 Water Vapor Transmission Rate (WVTR)

WVTR was determined using the method described in the literature.^21–23^ In summary, 10 mL of water was added to a beaker, which was then sealed with 2 × 2 cm PCL-P­(Arg) ENs. The beakers were weighed and maintained at 37 °C in a sealed chamber of the incubator. The beakers’ weight was recorded at consistent intervals throughout 8 days. An open bottle was used as the blank. The WVTR was calculated using eq 2 given below:

2 $$\mathrm{WVTR}=\frac{\Delta W}{tA}$$

where Δ*W* = weight difference, *t* = time in days, and *A* = test area.

### 2.6 *In Vitro* Drug Release

The *in vitro* drug release profile from the PCL-P­(Arg)/IBR ENs was studied at the physiological temperature of 37 °C, pH 5.5 (the endosomal pH of cancer cells and skin pH range of 4–5.5) and pH 7.4 (the physiological pH).^17,18^ The ENs were cut into 2 × 2 cm pieces and placed in various vials containing PBS (20 mL, pH 7.4 or pH 5.5). PCL-P­(Arg)/IBR ENs were maintained at 37 °C while being stirred at 250 rpm for 120 h. At specified time intervals, 200 μL samples were taken from the dissolution medium and substituted with 200 μL of fresh PBS buffer to ensure a consistent total volume. The samples were analyzed at 228 nm by using a UV–vis spectrophotometer (Thermo Fisher Scientific, ABD). The linear range for IBR was created using specific concentrations of IBR in DMSO:Distilled Water (1:9). The amount of IBR released was determined by using the IBR calibration curve. The cumulative drug release percentage from the PCL-P­(Arg)/IBR ENs was determined using the Eq 3:

3 $$P\left(\%\right)=\frac{P_{n}\times V_{0}\times \overset{n-1}{\underset{i=1}{\sum }}P_{i}\times V}{Q_{0}}\times 100$$

where *P* (%) = cumulative drug release, *P*_*n*_ = drug concentration at *n* time (μg/mL), *P*_*i*_ = drug concentration in the release medium (μg/mL), *V*_0_ = volume of release medium (mL), *V* = the volume of the withdrawn sample (mL), and *Q*_0_ = amount of loaded drug (μg).^24^

### 2.7 Study of the Drug Release Kinetics

The kinetic analysis of IBR release from PCL-P­(Arg)/IBR ENs utilized several mathematical models, such as zero-order (*Q* vs *t*), first-order (log­(*Q*_0_ – *Q*) vs *t*), Higuchi model (*Q* vs *t* ^1/2^), Hixson–Crowell (*Q*_0_^1/3^ – (*Q*_0_ – *Q*)^1/3^ vs *t*), and the Korsmeyer–Peppas exponential model (log *Q* vs log *t*). In this context, (*Q*) represents the quantity of drug released at time (*t*), *Q*_0_ represents the amount of drug remaining at time (0), while (*Q*_0_ – *Q*) signifies the amount of drug remaining at that time (*t*). The regression analysis was performed, and the model that best matches the release data was chosen based on the correlation coefficient (*R* ^2^). The Korsmeyer–Peppas model release exponent (*n*) was determined to identify the mechanism of drug release.^25–27^ The first 24 h of the release curves (early stage with linear part) were fitted to the kinetic models.

### 2.8 *In Vitro* Cell Viability Studies

The effects of PCL, PCL-P­(Arg), and PCL-P­(Arg)/IBR ENs on the viability of MCF-7 cells (human breast adenocarcinoma cell line, ATCC) were tested under *in vitro* conditions. MCF-7 cells were cultivated in Eagle’s minimum essential medium (EMEM), incubated at 37 °C with 5.0% CO_2_, and passaged twice a week. The ENs were cut into 2 × 2 cm pieces which deposited on the collector.^28^ Subsequently, both surfaces of the ENs were sterilized with UV light for 40 min and prepared for *in vitro* cell culture experiments. The 3-(4,5-dimethylthiazol-2-yl)-2.5-diphenyl tetrazolium bromide (MTT) test was used to assess the effect of ENs on cell viability. MCF-7 cells were seeded in 96-well microplates at a density of 5 × 10^3^ cells/100 μL and incubated for 24 h under standard culture conditions. After incubation, the medium in the wells was refreshed, and sterile ENs were transferred into the wells using forceps and then placed in the incubator for 24, 48, and 72 h under standard culture conditions. After the incubation periods, the samples and medium were removed from the wells, and 100 μL of the MTT solution (0.5 mg/mL) prepared in medium was added and incubated for 4 h. Following this incubation, 150 μL of DMSO solution was added to each well to facilitate the dissolution of formazan crystals in the orbital mixer. Absorbance measurements were performed at a wavelength of 570 nm using a UV–vis spectrophotometer, and the results were expressed as the percentage of cell viability. Untreated MCF-7 cells cultured under identical conditions without exposure to any ENs were used as the control group and defined as 100% viable. The relative cell viability of ENs-treated groups (PCL, PCL-P­(Arg), and PCL-P­(Arg)/IBR ENs) was calculated as the percentage of absorbance relative to that of the control group. All experiments were performed in five replicates, and statistical analysis was conducted using the Mann–Whitney U test.

## 3 Results and Discussion

### 3.1 Characterization of PCL-P­(Arg)/IBR ENs

Each parameter plays a critical role in the electrospinning process, where factors such as the applied voltage, polymer concentration, solvent characteristics, TCD, flow rate, temperature, and humidity must be meticulously fine-tuned. By optimizing these variables, one can unlock the potential for achieving the most favorable morphological properties in the resulting ENs.^29^ In our previous study, we optimized all electrospinning parameters to produce PCL ENs using a 12.5% (w/v) PCL polymer solution in FA:AC (3:7; v:v) solvent system, achieving optimal morphological properties.^19^ Consequently, PCL ENs were produced using a 12.5% (w/v) PCL polymer solution in FA:AC (3:7; v:v). To obtain PCL-P­(Arg) ENs, 10, 20, and 40 μg/mL of P­(Arg) were added to 12.5% (w/v) PCL solutions using the optimal electrospinning parameters. The SEM images demonstrated that all ratios produced nanofibers without beads (Figure 1A-C). Each SEM image was labeled (70 different points) with the ImageJ program to assess the distribution of the nanofiber diameters. The calculated diameter distributions of PCL-P­(Arg) ENs obtained with the addition of 10, 20, and 40 μg/mL P­(Arg) were found to be 405.08 ± 6.41, 340.21 ± 7.62, and 393.5 ± 8.78 nm, respectively (Figure 1D-F). Compared with other ratios, thinner and more uniform PCL-P­(Arg) ENs were obtained with a 20 μg/mL P­(Arg) addition. The enhanced conductivity of the polymer solution due to the polycationic P­(Arg) structure leads to an increase in charge within the solution, which facilitates the formation of the Taylor cone and results in a decrease in EN diameter, owing to the ample free charges on the surface of the polymer solution.^29,30^ As the concentration of P­(Arg) polymer increases (to 40 μg/mL P­(Arg)), the viscosity of PCL-P­(Arg) solutions increases, inhibiting the deformation of jets into ENs. Consequently, while all other parameters were maintained constant, the average diameter of the nanofibers expanded alongside the increase in P­(Arg) concentration.^31^ The literature also supports the finding that the diameter of ENs grows as the solution viscosity increases.^32^ Additionally, contact angle measurements were conducted to investigate how the contact angles of the obtained PCL-P­(Arg) ENs varied with the addition of 10, 20, and 40 μg/mL P­(Arg) to the PCL polymer solution (Figure 1G). The recorded contact angles of the PCL-P­(Arg) ENs for 10, 20, and 40 μg/mL P­(Arg) amounts were 104.90° ± 1.26, 100.60° ± 1.79, and 97.30° ± 1.4, respectively. Moreover, the time-dependent contact angle measurement of PCL-P­(Arg) ENs containing 20 μg/mL P­(Arg) was performed. It can be seen obviously that the water was completely absorbed by the PCL-P­(Arg) ENs at the end of 30 min (Figure 1I). The results show that the contact angle decreased as the P­(Arg) ratio increased due to the rising number of amine groups on the PCL-P­(Arg) ENs surface. Thus, the most suitable and uniform morphology for ENs was PCL-P­(Arg) ENs produced in the presence of 20 μg/mL P­(Arg). As a result, the production of PCL-P­(Arg)/IBR ENs was carried out with 20 μg/mL of P­(Arg).

**Figure 1:**
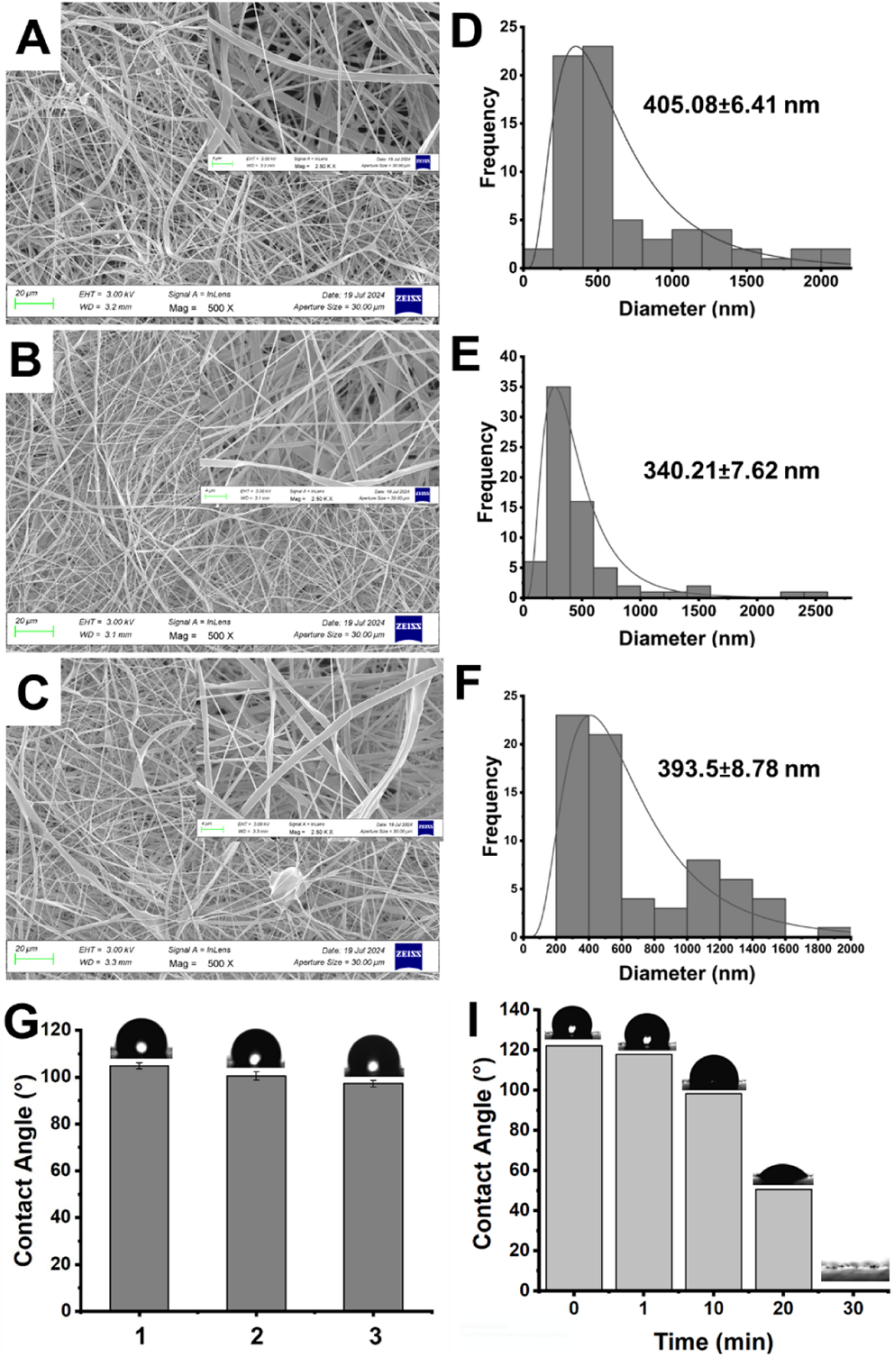
SEM images (500× magnification, scale bar represents 20 μm) of PCL-P­(Arg) ENs prepared using (A) 10 μg/mL P­(Arg), (B) 20 μg/mL P­(Arg), (C) 40 μg/mL P­(Arg) (inset shows SEM images (2500× magnification, scale bar represents 4 μm); nanofiber diameter distribution graphics of PCL-P­(Arg) ENs prepared using (D) 10 μg/mL P­(Arg), (E) 20 μg/mL P­(Arg), (F) 40 μg/mL P­(Arg), and contact angles of (G) 10 μg/mL (1), 20 μg/mL (2), and 40 μg/mL (3) P­(Arg) amount in PCL-P­(Arg) ENs, (I) Time-dependent contact angle measurements of PCL-P­(Arg) ENs containing 20 μg/mL P­(Arg) (Electrospinning process parameters: 8.0 kV (applied voltage), 10 cm (TCD), 1.0 mL/h (flow rate)).

Figure 2A displays the ATR-FTIR spectra of PCL and PCL-P­(Arg) ENs containing 20 μg/mL P­(Arg). The ATR-FTIR spectra of the PCL and PCL-P­(Arg) ENs show absorption bands at 2945 cm^–1^ and 2866 cm^–1^, which correspond to C–H stretching. The 1724 cm^–1^ and 730 cm^–1^ bands correspond to the −C=O functional groups. These peaks are characteristic of PCL.^25,33^ The ATR-FTIR spectra of PCL-P­(Arg) ENs did not show the expected amine groups of PCL-P­(Arg) ENs. This absence is likely due to the significant proportion of PCL in ENs production, which obscured the specific bands associated with P­(Arg). Therefore, we conducted an EDS analysis to identify N in PCL-P­(Arg) ENs containing 20 μg/mL of P­(Arg). The EDS spectra analysis of PCL ENs revealed that the elemental composition percentages for carbon (C) and oxygen (O) were 74.88% and 25.12%, respectively (Figure 2B). The C, N, and O elemental composition percentages for PCL-P­(Arg) ENs were 65.74%, 8.93%, and 25.32%, respectively (Figure 2C). The EDS analysis confirmed the presence of the N element on the surface of PCL-P­(Arg) ENs, demonstrating the successful integration of P­(Arg) into the PCL-P­(Arg) EN structure.

**Figure 2:**
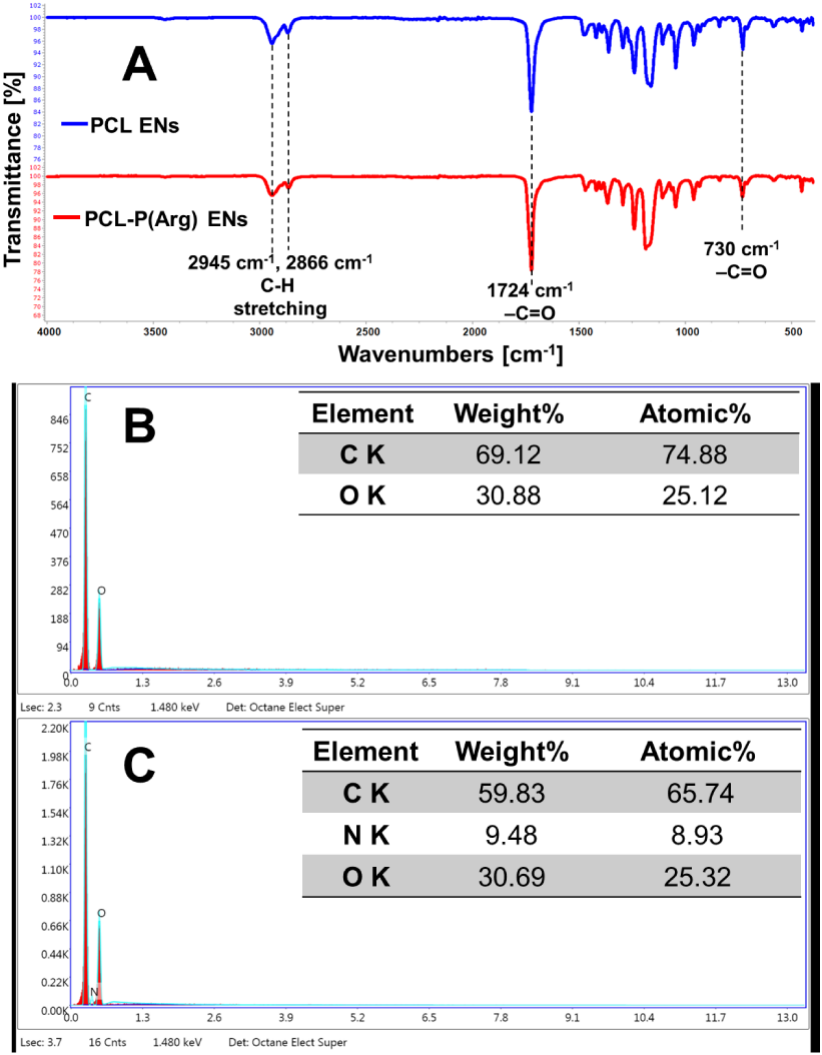
(A) ATR-FTIR spectra of PCL and PCL-P­(Arg) ENs and EDS spectra of (B) PCL ENs, and (C) PCL-P­(Arg) ENs.

The impact of incorporating IBR into PCL and PCL-P­(Arg) ENs on swelling capacity was examined to gain insights into the swelling behavior of the ENs in PBS (pH 5.5 and pH 7.4). The swelling ratios of PCL/IBR and PCL-P­(Arg)/IBR ENs are illustrated in Figure 3A-B. The inclusion of P­(Arg) significantly influenced the swelling ratio. The results indicated that the swelling rates after the incubation period (72 h) were 709.58 ± 31.31% and 1327.69 ± 91.23% for PCL/IBR and PCL-P­(Arg)/IBR, respectively. This indicates that PCL-P­(Arg)/IBR ENs swell 450% and 1093% more than PCL/IBR ENs at pH 5.5 and 7.4, respectively. P­(Arg) remains highly cationic because the guanidinium groups (−C­(=NH_2_^+^)–NH_2_) with a p*K*_a_ of approximately 13.8 are fully protonated at both pH 5.5 and 7.4.^34^ Consequently, the positively charged P­(Arg) chains repel one another, causing the fibers to expand. Also, strong guanidinium–water interactions through hydrogen bonding and electrostatic ion-dipole interactions further enhance water absorption, increasing swelling.^35^ However, the observed lower swelling at pH 5.5 compared to that at pH 7.4 can be attributed to the higher concentration of H^+^ ions and counterions at lower pH levels. This increase in H^+^ ions enhances charge screening among the P­(Arg) chains. This stronger charge screening reduces the electrostatic repulsion between chains, limiting EN expansion.^36^

**Figure 3:**
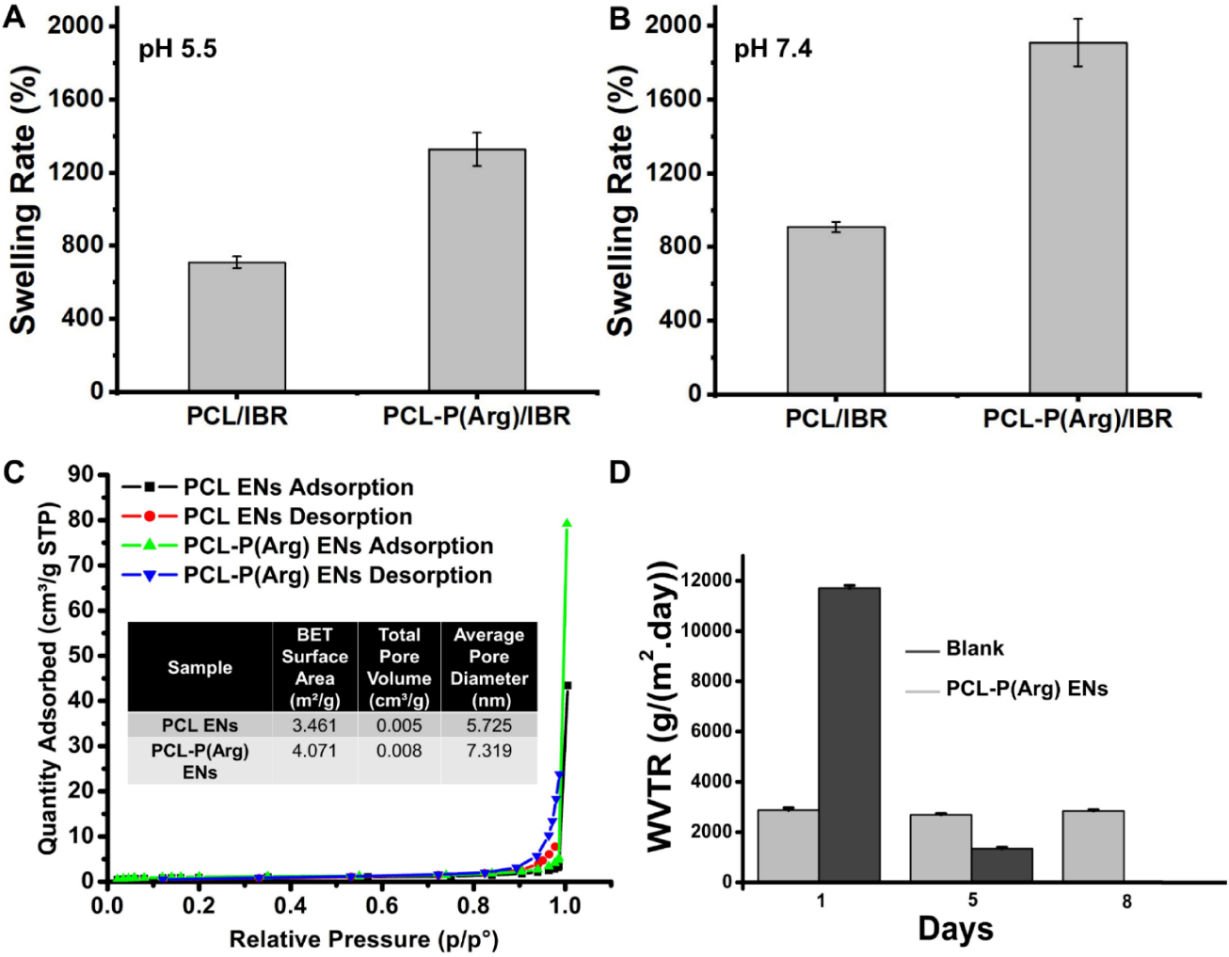
(A) The swelling percentages of PCL/IBR ENs and PCL-P­(Arg)/IBR ENs in PBS (pH = 5.5), *T* = 37 °C). (B) The swelling percentages of PCL/IBR ENs and PCL-P­(Arg)/IBR ENs in PBS (50 mM, pH = 7.4), *T* = 37 °C) at specific time intervals. (C) Isotherm linear plot of PCL ENs and PCL-P­(Arg) ENs (inset table presenting BET surface area, total pore volume, and average pore diameter of PCL/IBR ENs and PCL-P­(Arg)/IBR ENs) and (D) WVTR of PCL-P­(Arg) ENs and blank on days 1, 5, and 8 (All measurements were performed in three replicates).

The N_2_ adsorption–desorption isotherms of the PCL and PCL-P­(Arg) ENs are shown in Figure 3C. Both samples display Type IV isotherms according to IUPAC classification, characteristic of mesoporous materials.^37^ Additionally, the clear hysteresis loops indicate that capillary condensation occurs. The desorption branch of the PCL sample shows a gradual slope and a nonclosed loop resembling H3-type hysteresis—typically linked to slit-shaped pores—while the PCL-P­(Arg) ENs sample exhibits a more vertical and narrow hysteresis, suggesting H1-type behavior, which is usually associated with uniform cylindrical pores.^38,39^ Based on BET analysis, the PCL-P­(Arg) ENs displayed a higher specific surface area (4.071 m^2^/g) compared to that of the unmodified PCL ENs (3.461 m^2^/g), indicating that P­(Arg) incorporation contributed to the formation of a more porous and open EN network. Earlier research indicates that the BET specific surface areas of various ENs fall within the range of 0.6 to 33 m^2^/g.^40–42^ Additionally, the total pore volume, derived from N_2_ adsorption data at *P*/*P* ^o^ ≈0.99, increased from 0.005 cm^3^/g in PCL ENs to 0.008 cm^3^/g in PCL-P­(Arg) ENs. The average pore diameter from adsorption (4 V/A by BET) also increased from 5.725 to 7.319 nm, suggesting a broader and more accessible pore structure within the ENs, thereby confirming the mesoporous nature of PCL ENs and PCL-P­(Arg) ENs.^43–45^ The enhanced surface area and pore characteristics are strongly correlated with the observed swelling behavior and drug release performance. A more open fiber architecture facilitated greater water uptake, especially at pH 7.4, where the increased electrostatic repulsion between P­(Arg) chains further expanded the matrix.

The WVTR is a crucial factor in regulating the moisture levels in wounds. The low WVTR of wound dressings causes exudate accumulation, creating an inevitable risk of bacterial penetration and growth during healing.^46^ Research indicates that the ideal WVTR for wound dressings, which ensures adequate moisture retention and prevents fluid buildup at the wound site, ranges from 2000 to 2500 g/(m^2^·day).^47,48^ Figure 3D shows that the WVTR of PCL-P­(Arg)­ENs measured on days 1, 5, and 8 was calculated to be 2872.42, 2684.69, and 2844.17 g/(m^2^·day), respectively. The blank’s WVTR measured on days 1 and 5 was calculated as 11702.02 and 1339.14 g/(m^2^·day), respectively. For the blank group, measurement could not be performed after day 5 because the water in the container had evaporated entirely. According to the results, the calculated WVTR of PCL-P­(Arg)­ENs was far below than that of the blank group’s and very close to the acceptable range of 2000–2500 g/(m^2^·day). This may reduce the risk of wound dehydration and create a moist environment.

### 3.2 *In Vitro* Drug Release Behavior

While λ_max_ values of approximately 258–260 nm have been reported for IBR in various solvent systems, its UV–vis absorption is influenced by the solvent used.^49–51^ In the current aqueous medium containing DMSO, the most distinct absorbance maximum was observed at 228 nm, providing an optimal signal-to-noise ratio. To eliminate potential interference from DMSO, the same solvent mixture was used as the blank. Figure 4A illustrates that the IBR standard solution exhibited a maximum absorption at a wavelength of 228 nm. Consequently, this wavelength was selected for IBR quantification. The UV–vis absorbance of IBR solutions with varying concentrations was measured to establish a calibration curve for subsequent release studies (Figure 4B-C). As shown in Figure 4B, the absorbance intensity increased progressively with increasing IBR concentration, indicating concentration-dependent behavior. However, a deviation from linearity was observed above 40 μg/mL, most likely due to light scattering or detector saturation effects at higher concentrations. Therefore, the linear region between 0.05 and 40 μg/mL was selected for quantitative analysis (Figure 4C). The cumulative drug release profiles of PCL-P­(Arg)/IBR ENs in PBS medium at pH 5.5 (PBS) and pH 7.4 (PBS) are given in Figure 4D. According to the results, 28.63% of IBR release from PCL-P­(Arg)/IBR ENs was observed at the end of 24 h and 25.68% at the end of 120 h at pH 5.5. Although these data indicate that a rapid release (burst effect) occurred at the first stage, they remain within the expected range for EN-based drug delivery systems and demonstrate that IBR release continues at a more stable rate in the second stage.^5,52^ During the electrospinning process, the rapid evaporation of solvent may lead to partial surface localization of the drug molecules, particularly near the pore regions of the ENs.^53^ As previously noted, the swelling test performed at pH 5.5 revealed that PCL-P­(Arg)/IBR ENs swelled 450% more than PCL/IBR ENs. This significant swelling increases the porosity of the matrix, facilitating the entry of water and thereby initially accelerating the drug’s release from regions near the surface of ENs, which has the potential to create a burst effect.^54^ However, the release rate of 25.68% at 120 h remained comparable to that at 24 h, indicating that strong drug–EN surface interactions occurred within the system following the initial burst, thereby preventing a rapid release. Consequently, the IBR drug molecules located in the center of the PCL-P­(Arg) ENs were released slowly and sustainably at pH 5.5. PCL-P­(Arg)/IBR ENs exhibited release rates of 27.40% within 24 h and 33.53% at 120 h at pH 7.4, suggesting that the drug was initially released rapidly, displaying a slow and regular release pattern in long-term tests, similar to the release profile at pH 5.5. The greater release at pH 7.4 compared to pH 5.5 can be attributed to the fact that the PCL-P­(Arg)/IBR ENs swelled more at pH 7.4 than at pH 5.5 (709.58 ± 31.31% at pH 5.5 and 1327.69 ± 91.23% P­(Arg). Consequently, the PCL-P­(Arg)/IBR ENs became looser at pH 7.4, allowing more drugs to diffuse.

**Figure 4:**
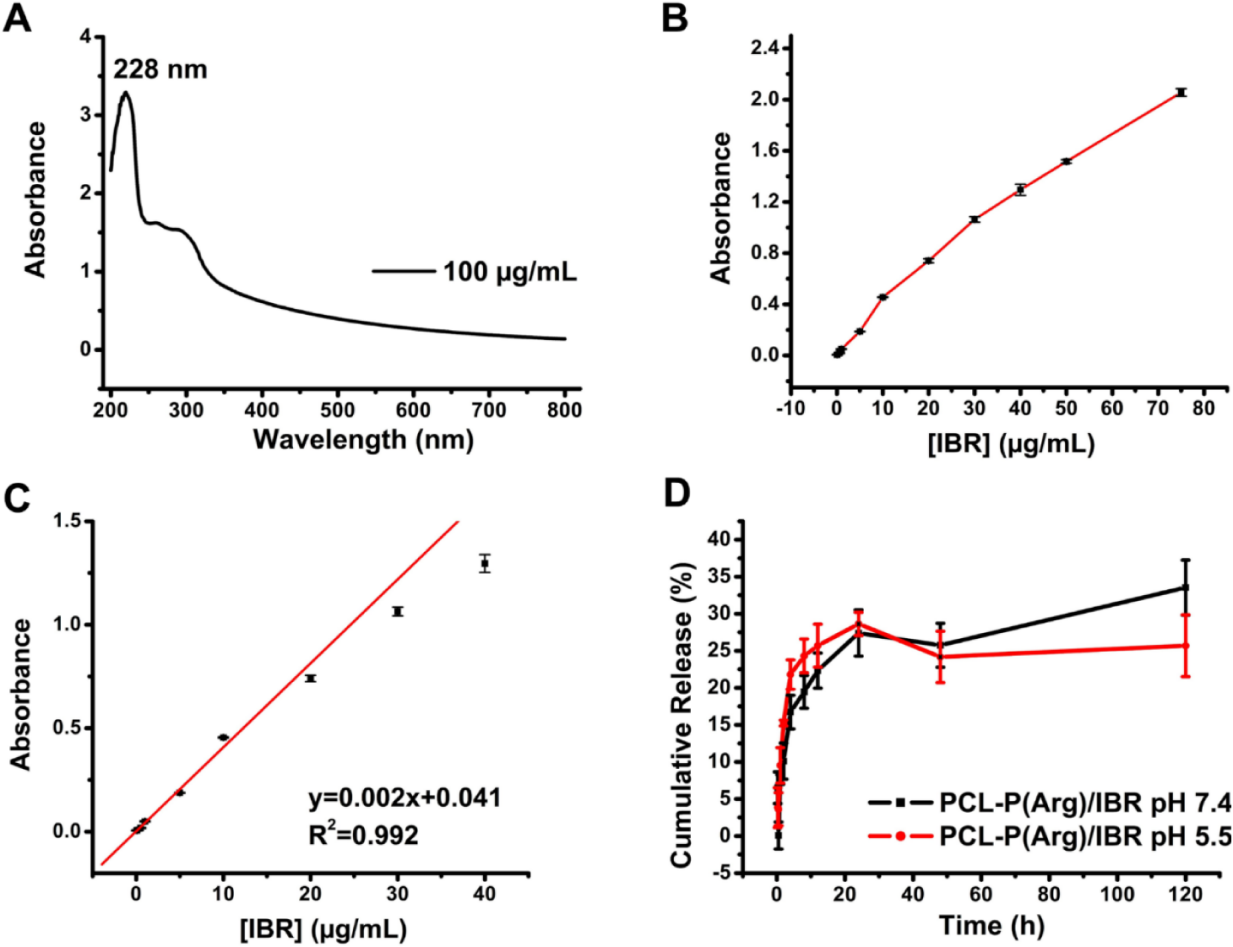
(A) UV spectrum of IBR standard solution (100 μg/mL) (UV–vis measurements were performed at 228 nm using DMSO:distilled water (1:9, v:v), (B) Working range for IBR (0.05–75 μg/mL), (C) Linear range for IBR (0.05–40 μg/mL), and (D) cumulative drug release profiles of PCL-P­(Arg)/IBR ENs in PBS at pH 5.5 and pH 7.4 (All measurements were performed in three replicates).

The free drug release profiles exhibited a notable slowdown after reaching 54.73% and 58.93% at 24 h in PBS release media at pH 5.5 and 7.4, respectively. At 120 h, the release stabilized at 50.89% and 65.65% at pH 5.5 and pH 7.4, respectively (Figure S1). The observation that the release amount for free drug release studies did not display a significant difference between pH 5.5 and 7.4 supports the notion that the primary limiting factor is the dissolution–diffusion balance. This indicates that the system reached equilibrium and no further diffusion could occur. Initially, the drug was released rapidly due to the high concentration difference between the interior and exterior. However, as the drug concentration in the external environment changed over time, the gradient diminished, leading to a lower release rate. IBR, a weakly basic compound (p*K*_a_ ≈ 3.7), exhibits greater solubility under acidic conditions due to increased protonation.^55^ At higher pH levels, reduced protonation markedly decreases its solubility, which can cause drug precipitation in the release medium and subsequently hinder the diffusion of free IBR molecules through the membrane.

The drug release kinetics from PCL-P (Arg)/IBR ENs were investigated by applying multiple mathematical models, including zero-order, first-order, Higuchi, Hixson–Crowell, and Korsmeyer–Peppas models (Figure 5A-E). Among these models, the Korsmeyer–Peppas model showed the best fit for both pH 5.5 and 7.4 conditions, with the highest *R* ^2^ values (0.924 for pH 5.5 and 0.960 for pH 7.4) (Figure 5F). A high *R* ^2^ value indicates strong agreement between the experimental release profiles and the theoretical predictions. Consistent with previous studies, ENs often show release behaviors that align well with the Korsmeyer–Peppas model due to the involvement of both Fickian diffusion and matrix swelling mechanisms simultaneously.^56–58^

**Figure 5:**
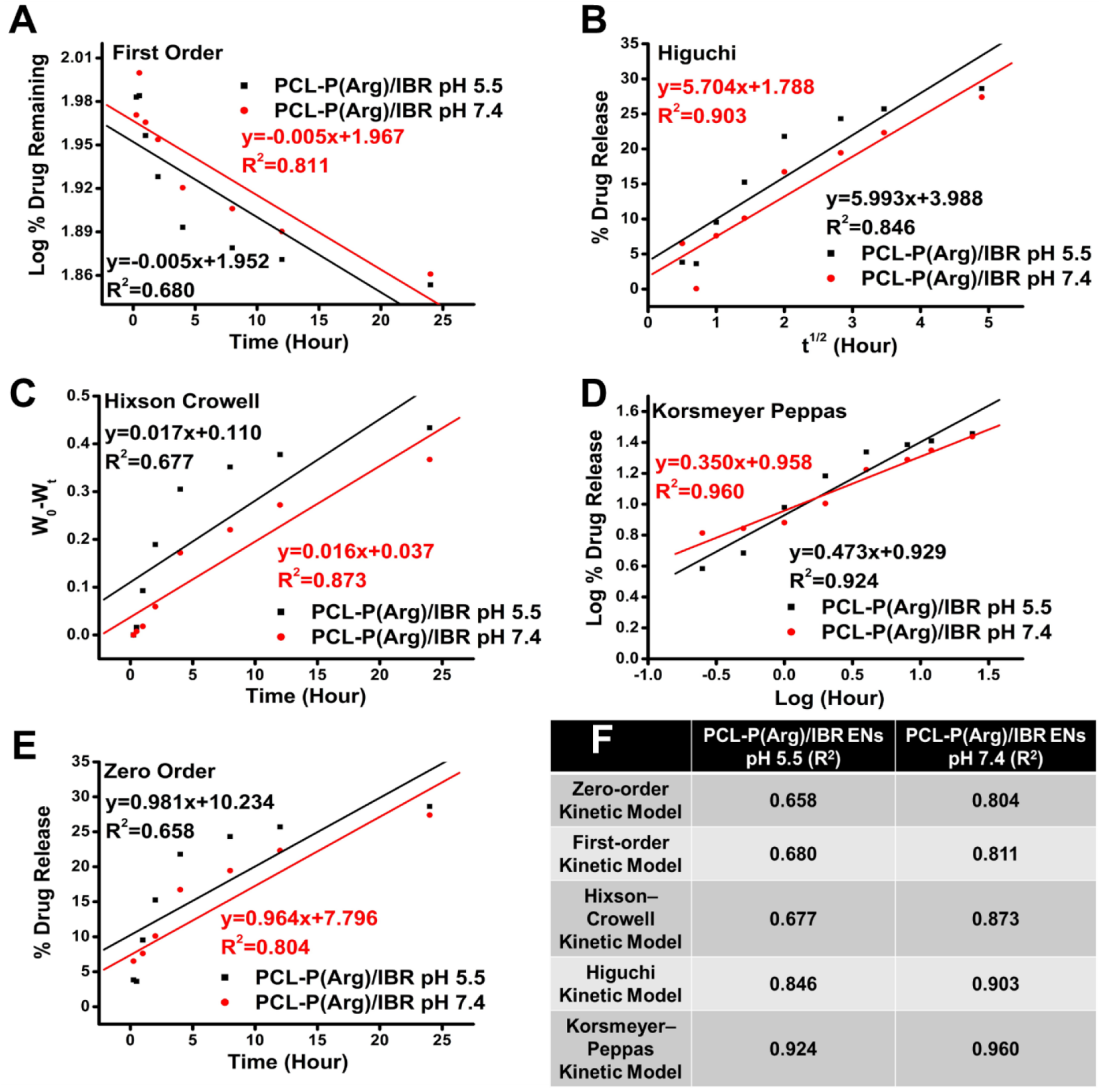
(A) First-order model kinetic release for PCL-P­(Arg)/IBR ENs at pH 5.5 and pH 7.4, (B) Higuchi model kinetic release for PCL-P­(Arg)/IBR ENs at pH 5.5 and pH 7.4, (C) Hixson–Crowell model kinetic release for PCL-P­(Arg)/IBR ENs at pH 5.5 and pH 7.4, (D) Korsmeyer–Peppas model kinetic release for PCL-P­(Arg)/IBR ENs at pH 5.5 and pH 7.4, (E) zero-order model kinetic release for PCL-P­(Arg)/IBR ENs at pH 5.5 and pH 7.4, and (F) Regression coefficient values of the different drug release models.

The diffusion exponent (*n*) derived from the Korsmeyer–Peppas fit further elucidated the underlying release mechanisms. According to Ritger and Peppas, when *n* < 0.45, the release follows a Fickian diffusion mechanism; an *n* value between 0.45 and 0.89 indicates anomalous (non-Fickian) transport; *n* = 0.89 corresponds to State II transport; and *n* > 0.89 represents super-State II transport.^59–62^ The calculated *n* values for the PCL-P­(Arg)/IBR ENs at pH 5.5 and 7.4 were 0.473 and 0.350, respectively. These results suggest that at pH 7.4, drug release was predominantly governed by Fickian diffusion, facilitated by the enhanced swelling and expanded pore network. The increase in specific surface area and pore size, confirmed by BET analysis, likely promoted faster water infiltration and drug diffusion at pH 7.4, which decreased the EN–drug interaction.^62^ At pH 5.5, it was observed that the anomalous transport mechanism, which encompasses both the swelling of ENs and diffusion, was effective throughout the release process. In diffusion-controlled release systems, swelling is affected by various factors. The chemical makeup, particularly the inclusion of hydrophilic groups, boosts water absorption, resulting in increased swelling.^56^ Environmental factors like pH and ionic strength are also important.^63^ At pH 5.5, the moderate swelling of PCL-P­(Arg)/IBR ENs limited the extent of the opening of the matrix. At the same time, the hydrophobic interaction between the drug and the ENs increased, slowing the drug’s diffusion. These observations confirm that the structural modifications introduced by P­(Arg) significantly influence both the swelling behavior and release kinetics of IBR in a pH-dependent manner.

### 3.3 *In Vitro* Cell Viability Studies

A model was used to assess the alterations in cell viability of the ENs on MCF-7 cells. MCF-7 cells were incubated with PCL, PCL-P­(Arg), and PCL-P­(Arg)/IBR ENs for 24, 48, and 72 h, and the effects on cell viability were tested by using the MTT assay. As shown in Figure 6, PCL, PCL-P­(Arg) ENs did not show any toxic effects on MCF-7 cells after 72 h, while IBR-loaded ENs caused a decrease in cell viability. The 24-h experiment indicated that the viability of cells treated with PCL, PCL-P­(Arg), and PCL-P­(Arg)/IBR was 98.17%, 93.47%, and 62.76%, respectively, according to the effect of ENs on cell viability. PCL-P­(Arg)/IBR ENs significantly decreased cell viability after 72 h compared to the other groups. The results indicated that PCL-P­(Arg)/IBR ENs were effective for the MCF-7 breast cancer cell line, which was one of the aims of this study.

**Figure 6:**
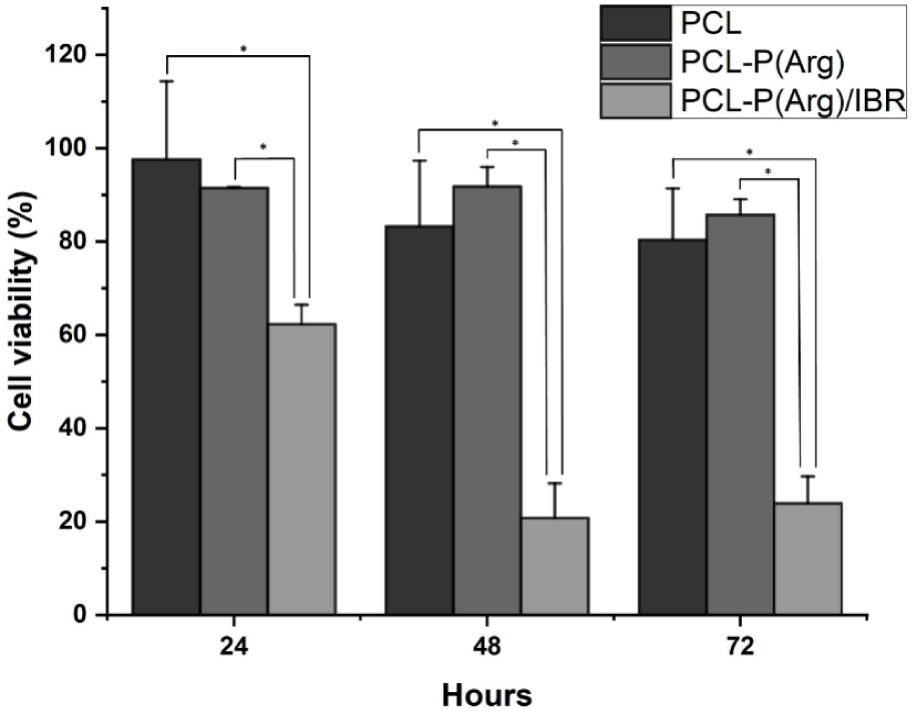
Cell viability (%) measured by MTT assay after 24, 48, and 72 h of incubation with PCL, PCL-P­(Arg), and PCL-P­(Arg)/IBR ENs. (Bars represent standard deviation, *n* = 5.) *P < 0.05.

## 4 Conclusion

This study demonstrates that the developed PCL-P­(Arg)/IBR ENs provide promising materials for the delivery of IBR in breast cancer treatment. Incorporating P­(Arg) into the PCL ENs improved the hydrophilicity and swelling behavior of the ENs, resulting in an enhanced release profile. The fabricated ENs exhibited sustained drug release over 120 h under *in vitro* conditions at pH 5.5 and 7.4, with release kinetics best described by the Korsmeyer–Peppas model. Moreover, *in vitro* cytotoxicity studies showed that the IBR-loaded PCL-P­(Arg) ENs exhibited a profound ability to decrease the viability of MCF-7 breast cancer cells while maintaining the PCL-P­(Arg) ENs’ biocompatibility, suggesting their potential in therapeutic applications. Notably, the time-dependent cytotoxicity results revealed a more pronounced reduction in cell viability at longer incubation times, particularly at 72 h, which can be attributed to the controlled and sustained release of IBR from the PCL-P­(Arg) EN matrix and prolonged drug–cell interactions. Despite these encouraging results, the present study is limited to *in vitro* characterization and cell-based assays, and *ex vivo* skin permeation as well as *in vivo* evaluations were not conducted. Therefore, future studies should focus on *ex vivo* skin diffusion experiments, *in vivo* validation, and further optimization of EN composition and drug loading to comprehensively assess the transdermal delivery efficiency and clinical relevance. Overall, these findings provide a solid foundation for the future development of nanofiber-based transdermal drug delivery systems for targeted breast cancer therapy.

## Supplementary Material



## Notes

Cumulative-free drug release profiles of PCL-P­(Arg)/IBR ENs in PBS at pH 5.5 and pH 7.4 (PDF)
