# Supplementary material for: In Vitro Testing of Ibrutinib-Loaded Electrospun Nanofibers for Potential Use as a Transdermal Patch Material
Source: ACS Omega. 2026 Feb 20;11(8):13845–54. doi: 10.1021/acsomega.5c12170 (PMC12961443; doi:10.1021/acsomega.5c12170)
Supplement: Supplementary file 1 [file ao5c12170_si_001.pdf]

Supporting Information

In vitro testing of ibrutinib-loaded electrospun nanofibers as a potential transdermal patch material

Hilal Fil[a], Ozan Yesiltepe[a,b], Simge Er Zeybekler[a], Ozge Kozgus[b], Sevinc Kurbanoglu[c], Emin Ilker Medine[b], and Dilek Odaci[a**]*

Email: dilekodaci.od@gmail.com , dilek.odaci.demirkol@ege.edu.tr

Table of contents

1.	Cumulative free drug release profiles in PBS at pH 5.5 and pH 7.4 (All measurements were performed in three replicates).	S2

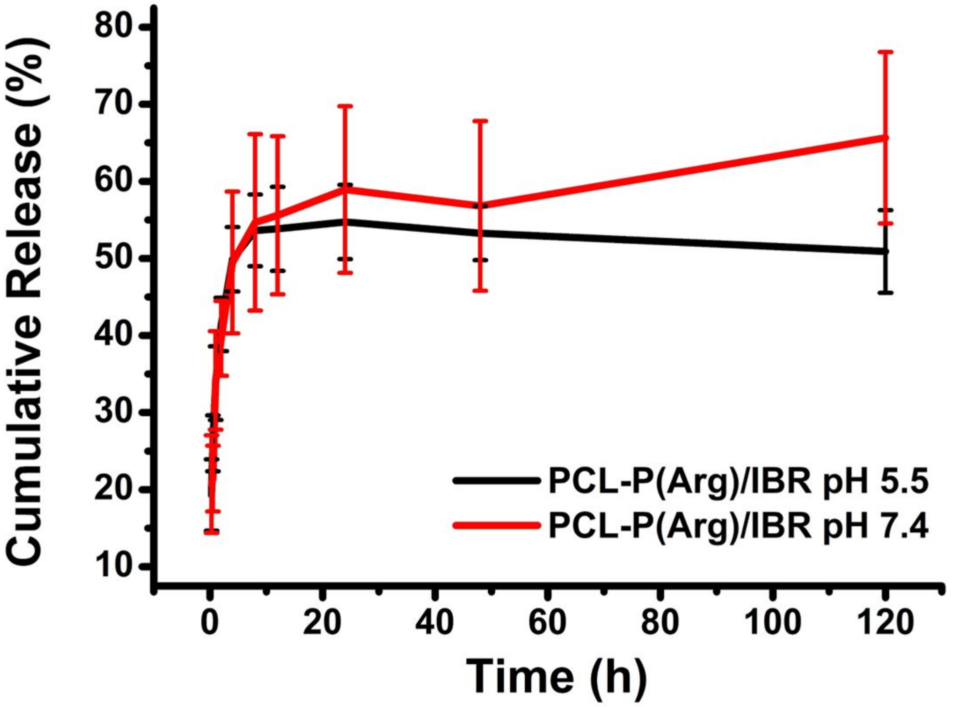

Figure S1. Cumulative free drug release profiles in PBS at pH 5.5 and pH 7.4 (All measurements were performed in three replicates).
